# Tools to Support Policy Decisions Related to Treatment Strategies and Surveillance of Schistosomiasis Japonica towards Elimination

**DOI:** 10.1371/journal.pntd.0001408

**Published:** 2011-12-20

**Authors:** Xiao-Nong Zhou, Jing Xu, Hong-Gen Chen, Tian-Ping Wang, Xi-Bao Huang, Dan-Dan Lin, Qi-Zhi Wang, Li Tang, Jia-Gang Guo, Xiao-Hua Wu, Ting Feng, Jia-Xu Chen, Jian Guo, Shao-Hong Chen, Hao Li, Zhong-Dao Wu, Rosanna W. Peeling

**Affiliations:** 1 National Institute of Parasitic Diseases, Chinese Center for Disease Control and Prevention, Shanghai, People's Republic of China; 2 Jiangxi Provincial Institute of Parasitic Diseases, Nanchang, People's Republic of China; 3 Anhui Provincial Institute of Parasitic Diseases, Hefei, People's Republic of China; 4 Hubei Provincial Institute of Parasitic Diseases, Wuchang, People's Republic of China; 5 Zhongshan School of Medicine, Sun Yat-Sen University, Guangzhou, People's Republic of China; 6 Diagnostics Research, London School of Hygiene and Tropical Medicine, London, United Kingdom; Institute of Tropical Medicine (NEKKEN), Japan

## Abstract

**Background:**

Appropriate diagnostics to monitor disease trends and assess the effectiveness and impact of interventions are essential for guiding treatment strategies at different thresholds of schistosomiasis transmission and for certifying elimination. Field validation of these assays is urgently needed before they can be adopted to support policy decisions of the national programme for control and elimination of schistosomiasis in P.R. China. We compared the efficacy and utility of different immunoassays in guiding control strategies and monitoring the endemic status of *S. japonicum* infections towards elimination.

**Methodology/Principal Findings:**

A cross-sectional survey was conducted in seven villages with different transmission intensities settings to assess the performance and utility of three immunoassays, e.g., an indirect hemagglutination assay (IHA_JX), an enzyme linked immunosorbent assay (ELISA_SZ), and a dot immunogold filtration assay (DIGFA_SH). 6,248 individuals aged 6–65 years old who gave consent and supplied their stool and blood samples were included for data analysis. Results showed that ELISA_SZ performed significantly higher sensitivity (95.45%, 95%CI: 92.94–97.97%) than IHA_JX (87.59%, 95%CI: 83.51–91.49%) and DIGFA_SH (79.55%, 95%CI: 74.68–84.41%), especially in subgroups with very low infection intensity. The specificity of ELISA_SZ, IHA_JX, DIGFA_SH in 6–9 year olds with occasional exposure was nearly 90%. DIGFA_SH performed the highest screening efficacy for patients among three assays with overall positive predicative value of 13.07% (95%CI: 11.42–14.72%). We found a positive correlation of antibody positive rate of IHA_JX with results of stool examination in age strata (r = 0.70, *P*<0.001). Seropositivity of IHA_JX in children aged 6–9 years old showed an excellent correlation with prevalence of schistosome infection in the seven communities (r = 0.77, *P*<0.05).

**Conclusions/Significance:**

Studies suggest that ELISA_SZ could be used to guide selective chemotherapy in moderate or low endemic regions. IHA_JX could be used to as a surveillance tool and for certifying elimination of schistosomiasis through monitoring children as a sentinel population.

## Author Summary

Immunodiagnostic assays are widely applied in the field to control schistosomiasis in P.R. China as the prevalence and infection intensity of schistosome infections decrease. Field evaluations are urgently needed before they can be adopted to support policy decisions of the national programme for the control and elimination of schistosomiasis in P.R. China. We carried out a large scale cross-sectional survey in field settings with different transmission situations to validate immunodiagnostic tools that can be used to formulate new schistosomiasis elimination strategy in P.R. China. Regarding stool examination as gold reference, the validity and screening efficacy of each immunodiagnostic kit were calculated and compared with each other. The association of the prevalence of schistosomiasis and antibody positive rates determined by immunoassays were analyzed using Pearson's correlation coefficient values. The study indicates that which test to use with the elimination strategy is dependent on the purpose of testing, the endemic status of community and the resources available. And more sensitive methods need to be explored and used to target infected individuals for treatment or to eliminate schistosomiasis.

## Introduction

Following great efforts by the Chinese government and professionals in public health, the People's Republic of China (P.R. China) has became one of the most successful countries in the world for schistosomiasis control [1]–[3]. Transmission had been interrupted in five provinces, Guangdong, Guangxi, Fujian, Shanghai, and Zhejiang by 1995 [1], [4]. The total number of infected individuals decreased by 90% through 50 years endeavour [3], [5], [6]. Due to changes in social and environmental factors including more migration people with changes in economic system, serious flood occurred in 1998 along the Yangtze River, resurgence of schistosomiasis became a public health problem in P.R.China in the beginning of the new century [3], [6], [7]. Since 2004, the central government reinforced the national control programme, and made schistosomiasis one of four priorities for infectious disease control in P.R.China. The new national goals aim to transition of the control strategy from morbidity reduction to transmission control: reduce prevalence of *S. japonicum* infection below 5% by 2008 (threshold of infection control) and below 1% by 2015 (threshold of transmission control) [6], [7]. To reach these goals, a novel control strategy was initiated in 2004 that aimed at implementation of integrated human and vector control measures to reduce the transmission of *S. japonicum* by blocking egg contamination of cattle and humans to infect snails [7], [8].

**Figure 1:**
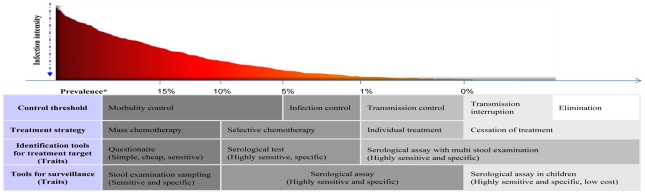
Chemotherapy strategies and tools for guiding treatment for schistosomiasis control in P.R.China. * Prevalence is calculated as the percentage of participants who were stool examination positive among the total number of people in the community.

Under policy of the national control programme, stages for schistosomiasis control are classified as morbidity control (prevalence over than 5% as defined by stool examination), infection control (prevalence of 1–5%), transmission control (prevalence lower than 1%), transmission interruption (no case detected in five years successively) and elimination (no case detected in another five years continuously after transmission was interrupted) in P.R. China [6]. Figure 1 shows current diagnostic approaches in relation to prevalence, treatment strategies and different control threshold towards schistosomiasis elimination in P.R. China. As transmission decreases, simple, affordable and accurate diagnostic tests are urgently needed for case detection, surveillance and assessment of the effectiveness of schistosomiasis control interventions. *S. japonicum* infection is usually determined by the Kato-Katz thick smear method and the miracidium hatching technique in P.R.China [9], [10]. However, stool examination is laborious and insensitive for monitoring in endemic areas characterized by very low prevalence and intensity of the disease, resulting in underestimation of the prevalence of infection in these regions [11]–[14]. Although they cannot be used to distinguish between current and past infection, immunodiagnostic assays based on antibody detection have been extensively applied in schistosomiasis control programme in P.R. China for years because of their advantages over parasitological tests including high sensitivity, rapid time to result, ease of use and ease of batching for population studies [15]–[17].

Three types of immunodiagnostic assays have been developed in P.R. China. Indirect hemagglutination assay (IHA) is currently the most widely used immunodiagnostic assay as a screening tool during World Bank Loan Project (WBLP) period and in national surveillance system on schistosomiasis in P.R. China [4], [18], [19]. Second, antibody-based enzyme linked immunosorbent assay (ELISA) was used to estimate the endemic status in the third nationwide sampling survey of schistosomiasis in P.R. China followed by Kato-Katz examination of seropositive individuals [6]. Third, rapid diagnostic assays such as dipstick dye immunoassay (DDIA) and dot immunogold filtration assay (DIGFA), were developed by Chinese laboratories for detecting cases infected with *S. japonicum* [20]–[22]. Although laboratory-based or epidemiological studies have been conducted to investigate the characteristics of immunoassays mentioned above for diagnosis of schistosomiasis [23]–[25], field validation of these assays have not been performed. These evaluations are urgently needed before they can be adopted to support policy decisions of the national programme for the control and elimination of schistosomiasis in P.R. China [26]. In this study, we sought to demonstrate that parasite density is low in settings with low prevalence of infection, making it necessary to switch from microscopy directly to serodiagnostic methods for surveillance. We will also explore, in low transmission intensity settings, the relationship between seropositivity and the prevalence of schistosome infection, as defined by stool examination, and with the intensity of transmission, as defined by egg count per gram of stool. As communities approach elimination, schistosome exposure in children as defined by antibody positivity can be used as surrogate of continued transmission. In this study, we explore the relationship between the rates of seropositivity in children and the prevalence of infection in the village.

Our study aims to validate immunodiagnostic tools that can be used to guide selective chemotherapy in moderate or low endemic regions to reduce the number of stool examinations required, to identify individuals for treatment in areas with very low endemicity, and to monitor the high risk population and certify the elimination of schistosomiasis.

## Materials and Methods

### Study design

A two-phase approach was used to assess utility of immunoassays to monitor *S. japonicum* infection. First the performance characteristics of existing and wild-spread used immunodiagnostic assays were assessed in the laboratory using a well-characterized serum panel of positive and negative samples. Second, tests with acceptable performance in phase one and easy to use, were selected for this study at field locations with different transmission intensity levels of *S. japonicum* infections. The results of the laboratory based evaluation of nine immunoassays have been reported elsewhere [27]. Three tests, an IHA assay (coded as IHA_JX), one DIGFA test (coded as DIGFA_SH) and an ELISA kit (coded as ELISA_SZ), were selected for the field trial based on their high sensitivity and specificity compared to the enzyme-linked immunoelectrotransfer blot assay (EITB) as a reference standard. All three kits detect serum antibodies against schistosome soluble egg antigen (SEA). The field studies were carried out between October to December, 2008. Before the field work and laboratory examination, experienced staff were selected and then trained through a training course by National Institute of Parasitic Diseases. A manual with standard operation procedure for each reagent was prepared and used for performing the test. 5% serum specimens and Kato-Katz slides were rechecked by national institute for quality assurance.

### Ethical statement

Written informed consents were obtained from all adult participants and the parents or legal guardians of children. Field-based researches were approved by ethics committees of National Institute of Parasitic Diseases, Chinese Center for Disease Control and Prevention. And individuals with positive stool examination results were treated with a single oral dose of praziquantel (40 mg/kg).

### Study areas and population

Field studies were conducted in seven villages, distributed in three provinces in the middle and lower reaches of Yangtz River in P.R. China with different estimated prevalence of schistosome infection based on stool examination. Villages included Caohui, Jingtou and Xinhua located in Jiangxi Province, Longshang, Tieguai and Yuye villages in Anhui province, and Hebei village was administrated by Hubei province. We invited all individuals aged from 6–65 years old to participate in our surveys. Over 50 grams of stool sample and 5 ml blood sample were collected from each participant who gave consent. Specimens were taken to local schistosomiasis stations for testing.

### Stool examination

Identification and quantification of *S. japonicum* eggs were carried out by the Kato-Katz thick smear method and the miracidium hatching technique in parallel [10]. Briefly, three slides (41.7 mg per smear) prepared from a single stool specimen were read 12–48 h after the initial preparation by two qualified technicians in a blinded manner. The number of eggs per slide was counted and recorded. The infection intensity of each individual was recorded as the number of egg per gram faces (EPG), calculated by adding up the number of eggs from all three slides and multiplying by eight to give EPG of stool. The left stool specimens were tested by miracidium hatching technique [10]. Observation was made at 4, 8, 12 hours after hatching. The result can be defined as negative if no miracidium was observed.

A patient is defined as positive if positive results were obtained with any parasitological technique. The infection intensity of individuals who were tested negative by Kato-Katz method but positive by miracidium hatching technique were regarded as one EPG. And participants were regarded as negative if negative results were obtained with both parasitological methods.

### Immunological tests

Blood samples were maintained in a vertical position for several hours to allow the spontaneous separation of the sera and red blood cells. The sera were removed into clear tubes and sealed. Serum specimens were tested by well trained technicians as following described according to the instructions supplied by the manufactures. Technicians performing the immunoassays were unaware of the results of stool examination and any other immunological test.

For ELISA_SZ, all serum specimens were diluted 1∶100 using the dilution liquid and transferred into the kit supplied microtiter wells. The incubation procedure, washing steps and detection steps were carried out according to the manufacturer's instructions. Absorbance was read at 450 nm zeroed by the reagent blank wells. For each run, positive and negative control sera were measured simultaneously. A positive result was defined as an optical density (OD) value greater than 2.1 times the OD value of the negative control serum provided by the kit [24].

For IHA_JX, 100 µl of normal saline was placed into the first well of the transverse line, whereas 25 µl was added into wells 2 and 3. Then, 25 µl of serum was added to the first well and thoroughly mixed. 25 µl of mixture was transferred to the second well and mixed as before while 75 µl of mixture in the first well was discarded. 25 µl of mixture in the second well was moved into the third well and mixed followed by discarding 25 µl of them. Thus, the concentrations of serum in the first, second and third wells were 1∶5, 1∶10 and 1∶20, respectively. Positive and negative control sera samples provided by the company were tested simultaneously on each plate. 25 µl sensitized red blood cell was placed into each well, shaken, kept at 37°C for 30 min. Observations were made by the naked eye. The titer in the test sera was recorded as one dilution before that which yielded a clear, sharp dark spot similar to that in the negative control wells. Titers were expressed as reciprocal values. Titers of ≥10 indicated a positive result [25].

DIGFA_SH was operated as following four steps: (1) Two drops of washing buffer from the buffer bottle was added to the well on the test box and penetrated the membrane completely; (2) 25 µl of serum was added to the same well and allow it to be absorbed completely; (3) The membrane was washed as step one followed by adding four drops of colloidal gold-labeled soluble egg antigen conjugate from the detecting bottle; (4) After the conjugate solution was absorbed completely, two drops of washing buffer were added to well to remove the unbound conjugate and then the result was read with naked eye immediately. The appearance of two red dots in the well indicated a positive reaction, and the appearance of a single red dot indicates a negative reaction [28].

### Statistical analysis

Only data from subject who gave consent and from whom both stool and blood samples were collected were used for analyses. Using stool examination as a gold reference standard, the prevalence of infection in each community was calculated as the percentage of participants who were stool examination positive among the total number of participants tested in that village. The consistency of results determined by stool examination and any immunoassay was measured by Kappa value.

Using stool examination as a reference standard, the sensitivity of each test was calculated as the percentage of participants who were seropositive among those who were positive by stool examination. Seropositivity represents exposure to schistosomes and is an aggregate of current and past infection. School aged children are sentinel population for new infection. The specificity of immunological testing in a subgroup population of 6–9 years old was calculated. Specificity of each immunoassay was expressed as the percentage of individuals who were seronegative among those who were stool examination negative. Furthermore, in low transmission intensity settings, it is useful to calculate the “positive predicative value” (PPV) of screening test by determining the percentage of those who were stool examination positive among those who were seropositive. This index gives a measure to evaluate the screening efficacy of immunoassays of what percentage of the schistosome exposed population is still shedding parasite. Comparisons of index between groups were analyzed by Chi-squared analyses.

The relationships between the prevalence and intensity of infection in each village, the antibody positive rate determined by the immunoassays and prevalence of schistosome infection were analyzed by Pearson's correlation analysis.

We judged a *P* value of less than 0.05 significant. All analyses were performed with SPSS (Statistical Products & Service Solutions) package for windows (SPSS Inc., Chicago, USA, version 13.0).

## Results

A total of 7,996 people in seven villages voluntarily participated in field survey. 10.27% (821/7996) of them didn't offer stool specimens while 6.64% (531/7996) didn't offer serum specimens. Of the remaining 6,644 persons, complete data were available on both fecal and serum specimens for 6,248 and were included in the analysis. This population, aged from 6–65 years, with a mean age of 38.53±17.76 years, was made up of 48.13% (3007/6248) men and 51.87% (3241/6248) women.

**Figure 2:**
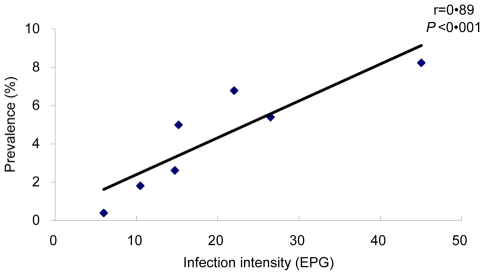
Correlation between prevalence and intensity of schistosome infection. r = Pearson's correlation coefficient.

The type of endemic areas and results of stool examination are shown in Table 1. The prevalence of *S. japonicum* infection in the seven villages, as defined by stool examination, ranged from 0.39%–8.23% with a geometric mean EPG (±S.D.) in the range of 5.98 (±7.90) to 45.04 (±4.98), indicating that these endemic areas had light infection intensity. Pearson's correlation coefficients (r) between the prevalence of schistosome infection and geometric mean EPG of positives was 0.89 (P<0.001), suggesting that an excellent correlation existed between the prevalence and intensity of schistosome infection at community level (Figure 2). All individuals infected with schistosomes or other parasites were given anthelminthes treatment.

**Table 1:** Characteristics of the villages included in the study and the results of stool examination.

Province	Village	Types of endemic areas	No. examined	Sex ratio(male/female)	Age(Mean±S.D.)	Prevalence of schistosome infection(% stool exam+)	Geometric mean EPG for infected(Mean ± S.D.)
Jiangxi	Xinhua	Marshland and lake region	922	0.98 (457/465)	34.34±20.15	4·99	15·18±6·24
Jiangxi	Caohui	Marshland and lake region	826	1.03 (418/408)	33.26±17.31	6·78	22·02±3·56
Jiangxi	Jingtou	Marshland and lake region	923	0.91 (440/483)	40.90±17.35	8·23	45·04±4·98
Hubei	Hebei	Marshland and lake region	886	0.99 (442/444)	31.96±18.72	1·81	10·48±7·26
Anhui	Longshang	Hilly and mountainous region	1032	0.99 (512/520)	39.52±15.37	0·39	5·98±7·90
Anhui	TieGuai	Hilly and mountainous region	843	0.70 (346/497)	48.25±12.18	2·61	14·73±3·18
Anui	Yuye	Marshland and lake region	816	0.93 (392/424)	41.78±16.48	5·39	26·50±4·43
Total			6248	0.93 (3007/3241)	38.53±17.76	4.23	23·74±5·06

The results of three immunoassays compared with those of stool examination based on field survey were shown in Table 2 and Figure S1. The Kappa values of immunological tests compared with stool examination ranged from 0.08–0.16, indicating very poor agreement between of immunoassays and parasitological detection (*P*<0.001).

**Table 2:** Performance characteristics of immunoassay compared with stool examination for the whole population.

Serological tests	Stool examination		Consistency with stool examination		Validity for cases detection		Screening efficacy	
	positive	negative	Kappa value	*P* value	Sensitivity[% (95% CI)]	*P* value*	PPV[% (95% CI)]	*P* value*
**ELISA_SZ**								
positive	252	2847	0·08	<0·001	95·45 (92·94–97·97)	<0·001	8·13 (7·17–9·09)	<0·001
negative	12	3137						
**IHA_ JX**								
positive	231	2254	0·10	<0·001	87·50 (83·51–91·49)	0.01	9·30 (8·15–10·44)	<0·001
negative	33	3730						
**DIGFA_SH**								
positive	210	1397	0·16	<0·001	79·55 (74·68–84·41)	NA	13·07 (11·42–14·72)	NA
negative	54	4587						

CI = Confidence interval, PPV = Positive predicative value, NA = Not available.

*P values were calculated by comparing with DIGFA_SH.

**Figure 3:**
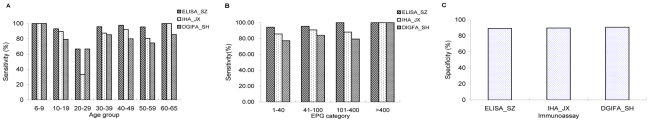
The sensitivities and specificities of three immunoassays and positives rates determined by each test. A: Sensitivities in age strata. B: Sensitivities calculated by EPG category. C: Specificity of each immunoassay in 6–9 years old subgroups.

For the whole population, ELISA_SZ and IHA_JX had sensitivity of 95.45% (95% CI 92.94–97.97%) and 87.50% (95% CI 83.51–91.49%), respectively, which were significantly higher than that of DIGFA_SH (79.55%, 95% CI 74.68–84.41%) (*P*<0.05). And also the sensitivity of ELISA_SZ was notable higher than that of IHA_JX (χ^2^ = 10.71, *P*<0.05). In stratified analysis, the sensitivity of ELISA_SZ and IHA_JX did not differ in age strata except in the group of 20–29 years old while DIGFA_SH presented significant difference in sensitivity among age groups (*P*<0.05) (Figure 3A). All assays showed sensitivities equal to or lower than 66.7% in the 20–29 years old group. These may be caused by the inadequate sample size of patients since there are only three stool positives in this age group and both of them were with EPG no more than 40. ELISA_SZ and IHA_JX also showed stable sensitivity among four intensity categories (*P*>0.05) while the sensitivity of DIGFA_SH increased with EPG notably (*P*<0.05) (Figure 3B). Chi-square analysis showed that the sensitivity of ELISA_SZ was significantly higher than those of IHA_JX and DIGFA_SH in subgroup with EPG in the range of 1–40 (*P*<0.05) while the sensitivity of these three assays didn't differ in other three infection intensity categories (*P*>0.05).

Since school-aged children could be used as sentinel population of new infection, the specificity of three immunodiagnostic tests was analyzed in 6–9 years old subgroup compared with stool examination (Figure 3C). ELISA_SZ, IHA_JX and DIGFA_SH showed specificity of 89.24%(95%CI: 87.71–90.77%), 89.73% (95%CI: 88.23–91.23%) and 90.71% (95%CI: 89.27–92.14%) respectively. And no significant difference in specificity was detected among these immunoassays in this subgroup of population (*P*>0.05).

The screening efficacy of each immunoassay to find the patients still shedding parasites among seropositives for the whole population was evaluated by calculating the PPV (Table 2). DIGFA_SH had the PPV of 13.07% (95%CI: 11.42–14.72%) which were significantly higher than that of other two assays (ELISA_SZ: 8.13% (95%CI: 7.17–9.09%) and IHA_JX: 9.30% (95%CI: 8.15–10.44) with *P* values less than 0·001 while no difference was detected in infectivity rate between ELISA_SZ and IHA_JX (*P*>0.05).

**Figure 4:**
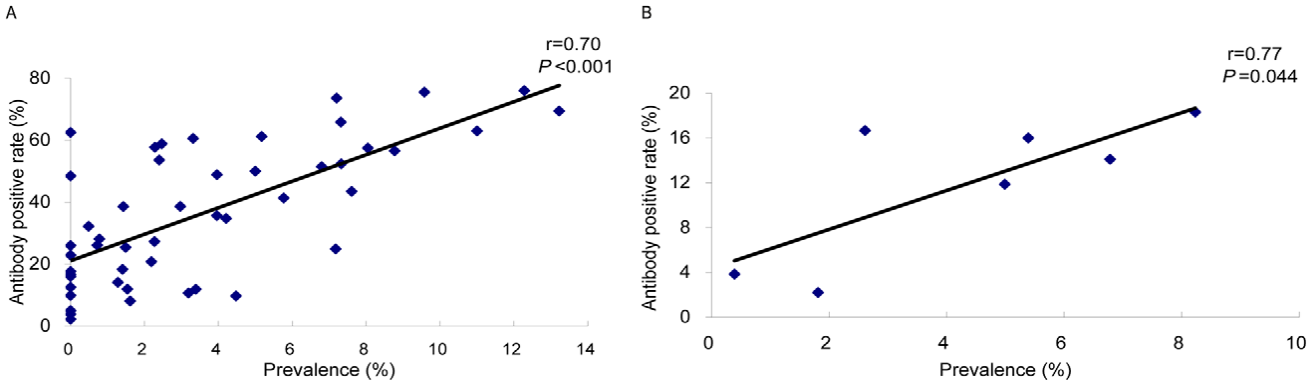
Correlation between antibody positive rate determined by IHA_JX and prevalence of schistosome infection. A: Relationship of antibody positive rate and prevalence of schistosome infection of communities in age strata. B: Relationship of antibody positives in group of 6–9 years old determined by IHA_JX and infection prevalence of villagers.

Antibody positive rates of whole population determined by ELISA_SZ, IHA_JX and DIGFA_SH were 49.60% ((252+2847)/6248), 39.77% ((231+2254)/6248) and 25.72% ((210+1397)/6248), respectively, which were significantly higher than the prevalence rate of schistosomiasis determined by stool examination (*P*<0.05). But the distribution tendencies of positives determined by each immunoassay and stool examination were consistent in age strata for the entire population. Pearson's correlation coefficient (r) between the prevalence of schistosome infection and antibody positive rates in age strata determined by IHA_JX was detected with r value of 0.70 (*P*<0.001), indicating the significant correlation between of them (Figure 4A). Antibody positive rate determined by IHA_JX in group of 6–9 years old as a sentinel population of schistosome infection also showed a significant correlation with the prevalence of infection amongst the villagers (r = 0.77, *P*<0.05, Figure 4B). The R-values for ELISA_SZ and DIGFA_SH were 0.44, 0.68 respectively, suggesting a lack of correlation.

## Discussion

The control of schistosomiasis was outlined as a series of consecutive phases changing from morbidity control to elimination of schistosome infection [2], [6], [29]. As the goals in different stage of schistosomiasis control varied, the diagnostic approach should be adjusted [26], [29], [30]. In P.R. China,with the ultimate aim to eliminate of schistosomiasis, a national programme with a comprehensive control strategy to reduce transmission had been implemented since 2004 [7], [8], [31]. During the implementation of national programme, appropriate diagnostic tools are important to yield accurate results to guide chemotherapy, monitor the endemic status and evaluate the efficacy of control intervention. It is well known that parasitological diagnosis for schistosomiasis is a poor prognosticator with insensitivity and increased operational time required in areas with low endemicity or intensity of infection [11], [13], [14], [32]. With many advantages over stool examination such as high sensitivity, simple operation and ease of use in the field, immunodiagnostic techniques had been developed and adopted into national programme of schistosomiasis control in P.R. China [6], [16], [18], [19]. Although many immunoassays are used in the field in P.R. China, little is known of serological tests that could be integrated into the elimination strategy, and used in the final stage of schistosomiasis control in P.R. China when prevalence and intensity of schistosome infection is low [3], [5].

In this study, we provided evidence that parasite density decreases as the prevalence of schistosome infection decreases, making it essential to switch to a more sensitive technique than microscopy for guiding treatment, surveillance and monitoring the impact of control interventions [26], [30]. As test-treat is the most cost-effective approach, immunodiagnostic assays was widely used for identifying target for treatment [15]. We found ELISA_SZ was more likely to be positive in individuals who had light infection intensity compared with IHA_JX and DIGFA_SH. This advantage was greater in subgroup with EPG low than 40. Considering results obtained by serologic tests were closer to the real status and sensitivity is given top priority for more efficient coverage with chemotherapy in low endemic areas [17], [29], [33], ELISA_SZ is appropriate as a screening tool for guiding selective chemotherapy with praziquantel in areas where prevalence of schistosome infection is higher than 1% in P.R. China with aim to reduce the number of infected cases, since praziquantel is a safe antischistosomal drug with few side-effects and toxicity [34], [35].

As transmission decreases especially lower than 1%, the treatment strategy needs to transit from selective chemotherapy to individual treatment to avoid over-treatment and praziquantel resistance, since studies have shown that praziquantel resistance against *S. mansoni* had been induced in lab and presented in humans in Senegal and Egypt [36]–[39]. Definition of individuals for treatment in endemic areas with such low prevalence and infection intensity needs more sensitive and specific diagnosis. Although polymerase chain reaction (PCR) based molecular methods had been reported for their higher sensitivity and specificity [40]–[42], disadvantages including dependence on expensive machines, complex operating procedure and long time reaction time, limit their widespread application for primary health-care settings. A two-step approach is the best choice until now with serology as the primary screening tool followed by stool examinations only for seropositive individuals [16]. The strategy and diagnostics used in the different stages of control in Figure 1 represent the current policy in China. ELISA_SZ assay is superior to other two assays as the screening tool for its high sensitivity and high throughput capacity. In addition, the high variation of geometric mean EPG of infected persons in our studies support that stool examination is inadequate when only one stool sample is detected in low endemic areas especially when the prevalence of schistosome infection was lower than 1% [11], [13]. Multiple stool examination or combination of other diagnostic methods for the positives of serological test would improve its sensitivity for individual treatment [29]. Our study also found that ELISA_SZ can miss some infected individuals especially with the egg count less than 100. This may only stimulate a relatively low antibody response which is not detectable by ELISA. A few individuals with low egg counts may be unlikely to spread disease further in the community. But to target infected individuals for treatment or to eliminate schistosomiasis, more sensitive methods need to be explored and used such as nested PCR.

Since specific antibodies against schistosome may last at least two years or even longer time in individuals who had past infection and cured after effective treatment [17], [25], [43], consistency of results determined by any immunological test and stool examination is very poor since the immunoassays also picked up antibodies from old exposures and it is not appropriate to calculate specificity of immunoassays directly by classifying seropositive–stool examination negative specimens as false-positives for the whole population. In our studies, the specificity of three immunoassays was calculated in subgroup of 6–9 years old since they could be regarded as sentinel population indicating new infection [44]. The results showed that these assays all performed high specificity about 90%.

Although the antibody positive rate determined by any immunological test was higher than the prevalence of schistosome infection determined by stool examination, we noted that the distribution tendencies of positives determined by immunoassays and stool examination were consistent in age strata for whole population [45]. The strong correlation of antibody positive rate determined by IHA_JX with prevalence of schistosome infection in age strata suggests that IHA_JX, which performed similar sensitivity in laboratory and field settings [27], could be used as a tool for surveillance and epidemiological study to analyze the epidemic characteristics of schistosome infection and estimate the distribution of high-risk infection population in communities [27], [32], [45].

Immunodiagnostic techniques had been shown as effective tools for disease surveillance through testing school-aged children in areas where transmission of schistosome was under control [44], [46]. In our work, the notable correlation between the antibody positive rate in children aged 6–9 years determined by IHA_JX and the prevalence of schistosome infection of villagers support this point and suggest that IHA_JX is an appropriate tool for schistosomiasis surveillance after the transmission of schistosome was under control (prevalence <1%) through measuring antibody response in school-aged children [47], [48]. Further investigation has recently started to explore how to identify the schistosome carriers in community through surveillance of school-aged children in area of low endemicity.

Furthermore, in low transmission intensity settings, screening assay which could identify more patients who are still shedding parasites among seropositives would be more efficient than doing stool examination [49], [50]. Here we calculated “PPV” to evaluate the screening efficacy of immunoassays. Our studies showed that DIGFA_SH was the most efficient screening assay among three assays with the “PPV” of 13.07% (95%CI: 11.42–14.72%). And with advantages over other two kinds of assays, such as quickness, easy operation and free of apparatus, DIGFA_SH is more appropriate for application in rural areas with limited resources for schistosomiasis control. Considering its relative low sensitivity especially for low infection intensity in our studies, DIGFA_SH needs to be improved to be more sensitive.

This study had several limitations. One limitation in our study is that the villages were not selected in a random manner and only one village with prevalence lower than 1% was included. The performance of immunoassays in other transmission control or transmission interruption areas might be different. The other limitation is that the three immunoassays are antibody-detection based tests which cannot distinguish history of exposure from active infection, it does not allow for estimation of the specificity of each immunoassay since the information of schistosome exposure history is difficult gained accurately. However, these assays could be used for diagnosis in occasionally exposed people such as travelers, migrants etc., and also could be used for surveillance in low-transmission or post-transmission settings. And also further studies focused on the cost-effectiveness of immunodiagnosis integrated into control strategies needs to be conducted.

In conclusion, our findings indicate that, despite good performance in laboratory setting, three immunoassays (ELISA_SZ, IHA_JX and DIGFA_SH) evaluated in our field trials perform differently in areas with different endemicity. The choice of which test to use with the elimination strategy is dependent on the purpose of testing, the endemic status of community and the resources available [29], [30]. ELISA_SZ is more suitable for guiding chemotherapy of schistosome infection in regions with moderate or low endemicity to reduce cases, and could be used as a screening tool for individual treatment followed by sensitive and specific definite diagnosis in areas after transmission is under control, and IHA_JX is easily applied in the risk assessment for epidemiological surveillance in endemic areas and certifying the elimination of schistosomiasis with monitoring the sentinel population while DIGFA_SH is a most efficient screening tools for chemotherapy in low endemic areas but its sensitivity needs further improved.

## Supporting Information

Figure S1 **Flowchart used for studies of diagnostic tests.**
(TIF)

Checklist S1 **STARD checklist for reporting of studies of diagnostic accuracy.**
(DOC)
