# Supplementary figures and images for: Tools to Support Policy Decisions Related to Treatment Strategies and Surveillance of Schistosomiasis Japonica towards Elimination
Source: PLoS Negl Trop Dis. 2011 Dec 20;5(12):e1408. doi: 10.1371/journal.pntd.0001408 (PMC3243709; doi:10.1371/journal.pntd.0001408)

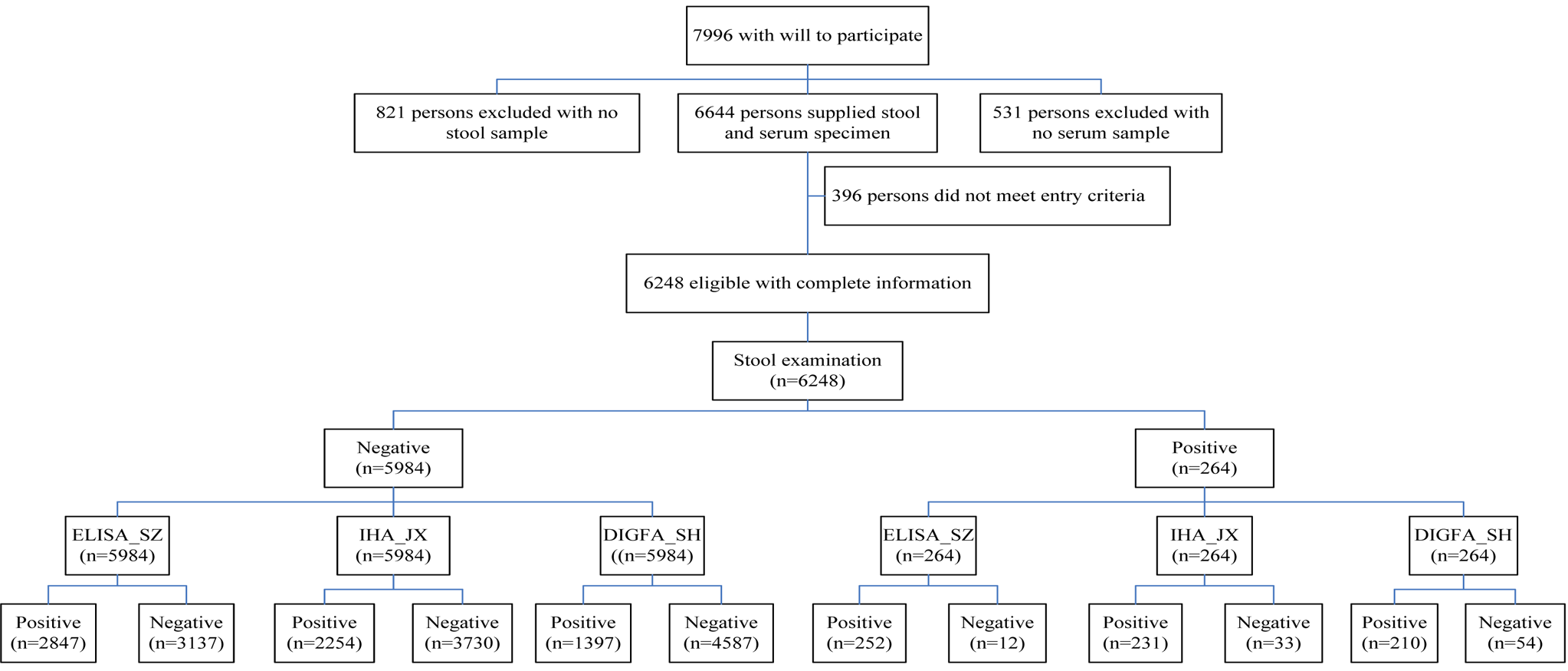

Supplement: Figure S1 — Flowchart used for studies of diagnostic tests. (TIF) [file pntd.0001408.s001.tif]
